# Prophylactic Role of Oral Melatonin Administration on Neurogenesis in Adult Balb/C Mice during REM Sleep Deprivation

**DOI:** 10.1155/2016/2136902

**Published:** 2016-08-07

**Authors:** Gabriela López-Armas, Mario Eduardo Flores-Soto, Verónica Chaparro-Huerta, Luis Felipe Jave-Suarez, Sofía Soto-Rodríguez, Iryna Rusanova, Dario Acuña-Castroviejo, Oscar González-Perez, Rocío Elizabeth González-Castañeda

**Affiliations:** ^1^Laboratorio de Microscopía de Alta Resolución, Departamento de Neurociencias, Centro Universitario de Ciencias de la Salud, Universidad de Guadalajara, Calle Sierra Mojada 950, 44340 Guadalajara, JAL, Mexico; ^2^Centro de Enseñanza Técnico Industrial (CETI) Campus Colomos, Ingeniería en Mecatrónica-Biomédica, Calle Nueva Escocia 1885, 44638 Guadalajara, JAL, Mexico; ^3^Departamento de Farmacobiología, Centro Universitario de Ciencias Exactas e Ingenierías, Universidad de Guadalajara, Boulevard Marcelino García Barragán 1421, 44430 Guadalajara, JAL, Mexico; ^4^Centro de Investigación Biomédica de Occidente, Instituto Mexicano del Seguro Social (IMSS), Calle Sierra Mojada 800, 44340 Guadalajara, JAL, Mexico; ^5^Instituto de Biotecnología, Centro de Investigación Biomédica, Parque Tecnológico de Ciencias de la Salud, Universidad de Granada, Avenida del Conocimiento s/n, Armilla, 18100 Granada, Spain; ^6^Laboratorio de Neurociencias, Facultad de Psicología, Universidad de Colima, Avenida Universidad 333, 28040 Colima, COL, Mexico

## Abstract

*Purpose*. The aim of this study was to assess the effect of melatonin in the proliferation of neural progenitors, melatonin concentration, and antiapoptotic proteins in the hippocampus of adult mice exposed to 96 h REM sleep deprivation (REMSD) prophylactic administration of melatonin for 14 days.* Material and Methods*. Five groups of Balb/C mice were used: (1) control, (2) REMSD, (3) melatonin (10 mg/kg) plus REMSD, (4) melatonin and intraperitoneal luzindole (once a day at 5 mg/kg) plus REMSD, and (5) luzindole plus REMSD. To measure melatonin content in hippocampal tissue we used HPLC. Bcl-2 and Bcl-xL proteins were measured by Western Blot and neurogenesis was determined by injecting 5-bromo-2-deoxyuridine (BrdU) and BrdU/nestin expressing cells in the subgranular zone of the dentate gyrus were quantified by epifluorescence.* Results*. The melatonin-treated REMSD group showed an increased neural precursor in 44% with respect to the REMSD group and in 28% when contrasted with the control group (*P* < 0.021). The melatonin-treated REMSD group also showed the highest expression of Bcl-2 and Bcl-xL as compared to the rest of the groups.* Conclusion*. The exogenous administration of melatonin restores the tissue levels of sleep-deprived group and appears to be an efficient neuroprotective agent against the deleterious effects of REMSD.

## 1. Introduction

Sleep deprivation (SD) is common event in the modern society that affects children, teenagers, adults, and old people [1, 2]. SD is a stressor phenomenon that has adverse consequences on brain function, especially when it occurs during the stage of rapid eye movement (REM) sleep. REM stage is considered essential for preserving context memory and facilitating long-term consolidation of visual discrimination tasks and emotional memory [3, 4]. In general, it is widely accepted that sleep facilitates brain restoration and tissue detoxification by removing oxidants produced during wakefulness [5]. SD induces the production of free radicals which causes oxidative damage mediated by imbalance between reactive oxygen species (ROS) and the endogenous antioxidant system [6, 7] in the brainstem and hippocampus [8, 9].

The subgranular zone (SGZ) of dentate gyrus (DG) in the adult hippocampus is a crucial target of SD effects because, in this, regions in new neurons (neurogenesis) are generated throughout life [10–12]. The incorporation of these newborn cells into the existing neuronal circuitry has been associated with the optimization of memory processes and cognitive functions [13]. Experimental evidence indicates that SD produces a significant reduction in the number of new neurons in the SGZ, which may impair learning and memory performance [10, 14–22].

Circadian rhythms are induced by the daily light-dark cycle [11, 12] and are regulated by the pineal gland, a small endocrine gland that controls the rhythmic production of melatonin. This hormone mediates its effects through the cell membrane receptors MT1 and MT2 [23, 24] and regulates antioxidant enzymes [25, 26] by switching on/off intracellular signaling cascades [27, 28], as well as by scavenging oxygen free radical [29–33]. In addition, melatonin promotes neurogenesis under diverse conditions, such as ovariectomy, pinealectomy, aging, or circadian disruption [17, 34–40]. In this study, we used a prophylactic administration of melatonin for 14 days (before and during 96 hours of REMSD) to determine if melatonin exerted a protective effect in neural precursor cells. Our results show a significant improvement in the survival of neural precursors in the melatonin-treated REMSD group with respect to the REMSD untreated group. These findings were associated with an increased expression of antiapoptotic proteins Bcl-2 and Bcl-xL in the melatonin-treated REMSD group.

## 2. Materials and Methods

### 2.1. Animals

50 adult male mice Balb/C (23–26 g) were used for this study. Animals were maintained in a temperature-controlled environment on a 12 h light-dark cycle with light on at 08:00 h, with room temperature of 24°C ± 2, and with free access to food and water. Animals started melatonin and luzindole (once daily) treatment for 14 days before 96 h REMSD and during SD until the end of the experiment. Mice were randomly distributed in five groups (*n* = 10 mice per group): (1) control, (2) REMSD, (3) melatonin + REMSD, (4) luzindole + melatonin + REMSD, and (5) luzindole + REMSD. For HPLC and Western Blot analyses, five animals per group were sacrificed by decapitation to obtain fresh and unfixed tissue. The rest of the animals received a single injection of BrdU (100 mg/kg) 2 hours before sacrifice by intracardiac perfusion. Sacrifices were made immediately after the end of REMSD exposure. All the experiments were designed to minimize the number of animals used in every experiment. The procedures described herein were carried out in accordance with the regulation indicated by the Ethics Committee of the University of Guadalajara and following NIH regulations.

### 2.2. Drug Preparation for Continuous Administration and Intraperitoneal Injection

#### 2.2.1. Melatonin Treatment

Melatonin (Sigma-Aldrich Co., St. Louis, MO, USA) was prepared and changed every third day in a minimum volume of 1% absolute ethanol and tap water to reach a concentration of 10 mg/kg of body weight (b.w.) per day [40, 41]. During treatment, melatonin was placed in a drinking water and protected from direct light with aluminum foil. Melatonin chronic administration is well supported by Ramírez-Rodríguez et al. and Silva et al. [35, 36, 42]; they administered melatonin as far as 9 months with no side effects reported like drowsiness.

#### 2.2.2. Luzindole Treatment

Luzindole antagonist MT1 and MT2 melatonin receptors [23] (Santa Cruz Biotechnology, USA) were prepared daily in a minimum volume of ethanol 1% and sterile water for intraperitoneal injections. Luzindole was used at 5 mg/kg of b.w. per day [43]. Luzindole was administered once daily with an intraperitoneal injection at 17:30 h. For the group with both melatonin and luzindole, melatonin was given by an oral cannula 30 minutes after injection of luzindole. With respect to luzindole treated mice we do not observe any unusual behavior.

### 2.3. REM Sleep Deprivation

To select REMSD time, we analyzed corticosterone serum levels at various time points of SD: 24 h, 48 h, 72 h, and 96 h and we found basal corticosterone levels at 96 h of SD [44]. Mice were deprived of REM sleep using the inverted flowerpot technique [45, 46]. Briefly, REM sleep-deprived mice were placed on inverted flowerpots 2.5 cm in diameter and 20 cm in height surrounded by water in a 180 cm diameter tank. Water level was within 7 cm of the top of the flower pot. The control group was maintained in a plexicage in the same room with similar conditions but out of the water tank since it has been demonstrated that large platforms in tanks make animals lose 80% of sleep time [47]. This paradigm suppresses REM sleep since the decrease in muscle tone during this phase makes animals fall into the water. This technique is well accepted as a model for depriving rodents of REM sleep without the requirement for electroencephalography (EEG) monitoring [48]. Mice inside the container were always able to reach either food, melatonin bottle, or tap water according to each experimental group. Water temperature was controlled at 23 ± 3°C.

**Figure 1:**
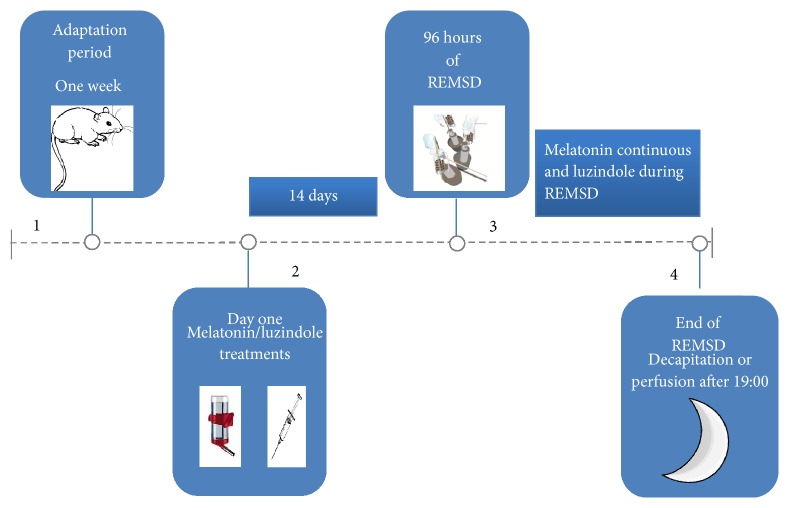
Timeline. Square 1. Animals were kept one week in the adaptation stage. Square 2. Animals begin with melatonin/luzindole treatments for 14 days. Square 3. 96 h REMSD. Square 4. REMSD is finalized and sacrifice by decapitation or intracardiac perfusion is performed on animals.

The application of the melatonin, luzindole, and REMSD is shown in the time points diagram (Figure 1).

### 2.4. Tissue Samples

Once the period of REMSD was completed, after 19:00 pm, some of the animals were decapitated. Immediately after that, the brain was dissected on ice to obtain the hippocampal tissue. Brain tissue samples were stored at −80°C for further analysis. Mice for immunofluorescence were deeply anesthetized with a lethal dose of pentobarbital and were transcardially perfused with phosphate buffer (PB) of 4% paraformaldehyde. Brains were fixed for 24 h in this same solution. Afterwards, brain specimens were cut at 35 *µ*m using a vibratome (Leica, Microsystems) starting from rostral Bregma, −1.34, to caudal Bregma, −2.92 mm [49]. Tissue storage was done in PBS plus 0.3% sodium azide until used.

### 2.5. Determination of Melatonin Levels in Hippocampal Tissue

Hippocampal melatonin levels were measured in all groups. After hippocampi defrost (20 mg each one approx.) each tissue was sonicated in 400 *µ*L of 0.1 M phosphate buffer containing 0.15 M NaCl, pH 7.4, and centrifuged at 3000 ×g for 10 minutes at 4°C. An aliquot of the supernatant was frozen at −80°C for protein determination. Another supernatant aliquot (320 *µ*L) was mixed with 1 mL chloroform and 320 *µ*L of 0.1 M acetate-acetic buffer, pH = 4.6, shaken for 20 minutes, and centrifuged at 9000 ×g for 10 minutes at 4°C. The organic phase was washed with 0.1 N NaOH solution. Five hundred microliters of this mixture was evaporated to dryness in a concentrator 5301 (Eppendorf, AG). The residue was dissolved in 100 *µ*L of an HPLC mobile phase and measured with HPLC Shimadzu (Shimadzu Corporation, Duisburg, Germany), with a 4.6 × 150 mm reverse-phase C18 Sunfire Column (Waters Corporation, Milford, MA, USA) [50]. After stabilizing the column with the mobile phase, 40 *µ*L of each sample was injected onto the HPLC system. The mobile phase consisted in 0.1 M sodium phosphate, 0.1 mM ethylenediaminetetraacetic acid, and 25% acetonitrile, pH 5.2, at a flow rate of 1 mL/minute. A standard curve for melatonin was constructed in the range between 200 and 1500 pg/mL of melatonin standard. 5-Fluorotryptamine was used as the internal standard. The fluorescence of the samples was measured with a fluorescence detector (Shimadzu, RF-10A XL, Shimadzu Corporation), with excitation/emission wavelengths of 285/345 nm, respectively. Melatonin concentration was expressed in pg/mg of tissue protein.

### 2.6. Antibodies

All antibodies were diluted in PBS 0.1 M containing 10% goat serum. The primary antibodies for immunofluorescence used in this study were monoclonal rat anti-BrdU MCA2060 (Abd Serotec, Bio-Rad Laboratories, Inc., 1 : 400.) monoclonal mouse and anti-nestin MAB353 clone rat 401 (Millipore, Merck KGaA, Darmstadt, Germany 1 : 100). Secondary antibodies were as follows: Alexa Fluor 488 for BrdU label and Alexa Fluor 594 for nestin label (Invitrogen*™*, Thermo Fisher Scientific Inc., 1 : 1000). For Western Blot, we used the following: anti-bcl2 rabbit polyclonal (Novus Biologicals, LLC, USA, 1 : 1000), anti-Bcl-xL mouse monoclonal (Sigma-Aldrich Co., St. Louis, MO, USA, 1 : 1000), and anti-*β*-actin (Santa Cruz Biotechnology, USA, 1 : 2500). Secondary antibodies included biotinylated anti-rabbit and anti-mouse (Vector Laboratories Ltd., Southfield, MI, 1 : 1000).

### 2.7. Immunofluorescence

For immunofluorescence double-labeling for BrdU and nestin antibodies, tissues were pretreated with 2 N HCL for 10 min at 37°C followed by 10 min in boric acid 0.1 M (pH 8.5) at room temperature. Subsequently, tissues were washed in PBS-Triton-X-100 0.03% three times for 5 minutes and blocked with goat serum 10%. Primary antibodies were incubated overnight at 4°C. After three times, of 5 min washing, they were incubated with secondary antibodies for 1 hour at room temperature. Finally, tissues were washed exhaustively and mounted with Vectashield Mounting Medium with DAPI (Vector Laboratories Ltd., Southfield, MI) and quantified in a fluorescence microscope Carl Zeiss MicroImaging GmbH with Colibri lighting system and Carl Zeiss AxioVision SE64 Rel Software (Göttingen, Germany).

### 2.8. Quantification of BrdU/Nestin Labeled Cells

One parallel series of every sixth free-floating brain section was used for fluorescence double-immunolabeling of BrdU and nestin. Progenitor cells were counted using a 40x objective throughout the rostrocaudal extent of the subgranular zone (SGZ) which is structurally located between granular cell layer and hilus of the DG of both sides. Counting was done as described earlier, with a modified optical dissector method. The cells appearing in the uppermost focal plane were omitted to avoid oversampling [51]. The resulting numbers were multiplied by six to obtain the estimated total number of BrdU and nestin labeled cells per SGZ. All quantifications were performed blinded to group assignment.

### 2.9. Western Blot Analysis

Immediately after 96 h of SD, mice (*n* = 5 for each group) were sacrificed by decapitation and hippocampus samples were dissected and immediately frozen to be stored at −80°C until use. Then, hippocampus samples were homogenized in lysis buffer (PBS 0.1 M and protease inhibitor cocktails), and the total protein concentration was determined using the Lowry protein determination (Bio-Rad Laboratories, Inc.). Samples were loaded on 12% SDS-polyacrylamide gels, separated by electrophoresis, and then transferred to nitrocellulose membranes (Amersham GE, Little Chalfont, UK). Immunodetection was performed with diaminobenzidine tetrahydrochloride method (DAB, Sigma-Aldrich, St. Louis, MO). Membranes were blocked with 5% nonfat milk for 1 h and then incubated with primary antibodies overnight at the indicated dilutions: anti-Bcl-2, anti-Bcl-x, and anti-*β*-actin and with secondary antibodies biotinylated for 2 hours at room temperature. Afterwards, they were treated with avidin-biotin complex, 1 : 100 (Vector Laboratories Ltd., Southfield, MI), and developed with DAB. To normalize the signals of Bcl-2 and Bcl-xL, the corresponding signals of *β*-actin were measured on the same blots. We analyzed densities with ImageJ software (NIH, USA). Mann-Whitney *U* tests were performed to compare differences between expression levels in the different groups for each protein.

## 3. Statistical Analysis

Analyses were carried out with SPSS version 20 and Graphpad Prism version 5.01 software. Results are presented as means ± SD. Statistical analyses of the data were performed using the Kruskal-Wallis nonparametric test and Mann-Whitney *U* test. The level of significance was set at *P* < 0.05.

## 4. Results

Melatonin and luzindole administration were well tolerated and no side effects were observed throughout the study.

### 4.1. Melatonin Concentration Varies in Hippocampus Homogenate under REMSD Exposure

**Figure 2:**
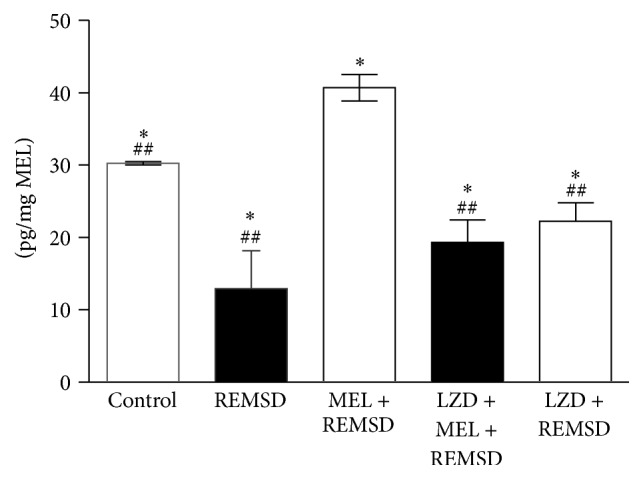
Hippocampal melatonin under REMSD. Bars express means ± standard deviation. Statistical analysis showed significant differences between groups: control group versus LZD + MEL + REMSD; only REM sleep deprivation and LZD + REMSD (^##^ *P* < 0.046) Mann-Whitney *U* test. Furthermore, the MEL + REMSD group revealed a higher concentration of melatonin in comparison to the control, REMSD, LZD + MEL + REMSD, and LZD + REMSD groups (^*∗*^ *P* < 0.05); Mann-Whitney *U* test. REMSD: REM sleep deprivation, MEL: melatonin, and LZD: luzindole.

This far, there are no reports that determine melatonin concentration in hippocampus homogenates under REMSD conditions in wild-type animals. Our results show that the control group (non-sleep-deprived animals) had statistically significant differences in the concentration of melatonin (30.62 ± 0.23 pg/mL, *P* < 0.04) with respect to the REMSD group (12.94 ± 5.2 pg/mg), the group luzindole + melatonin + REMSD (19.37 ± 3.05 pg/mg), and the group luzindole + REMSD (22.27 ± 2.5 pg/mg) (Figure 2). Animals that received oral melatonin showed higher concentrations of melatonin (40.71 ± 1.8 pg/mg, *P* < 0.05) and presented significant differences when compared to controls, the REMSD group, and the luzindole groups (luzindole + melatonin + REMSD and luzindole + REMSD).

### 4.2. Melatonin Protects Survival of Neuronal Precursors in SGZ

**Figure 3:**
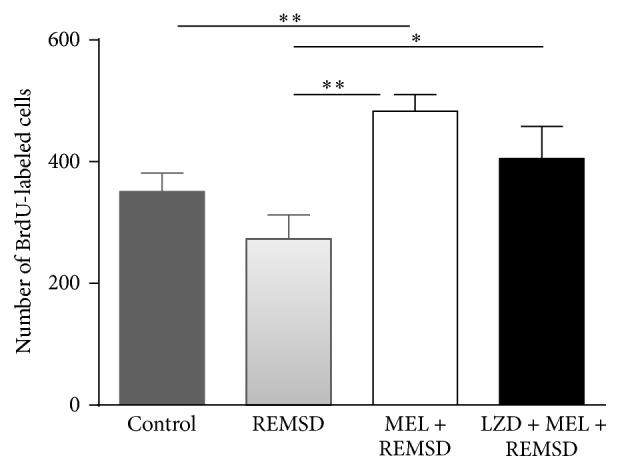
Number of BrdU label cells in dentate gyrus of hippocampi tissues. Bars express means ± SD. (*∗*) Statistical differences were found for the control group versus MEL + REMSD and REMSD versus MEL + REMSD groups (^*∗∗*^ *P* < 0.021). A statistical difference was found for REMSD versus LZD + MEL + REMSD groups (^*∗*^ *P* < 0.043). Mann-Whitney *U* test.

Melatonin has been shown to promote neurogenesis in both* in vitro* and* in vivo* experimental conditions, but these effects have not been investigated in REMSD [22, 52–54]. The quantification of BrdU-positive cells for the control group (351 ± 60 cells per SGZ) showed no significant differences when compared to the REMSD group (273 ± 79 cells per SGZ). In contrast, the melatonin + REMSD group (483 ± 55 cells per SGZ, *P* < 0.021) showed a statistically significant difference when compared to the REMSD (273 ± 79 cells per SGZ) and control group (351 ± 60 cells per SGZ). This change represents 44% and 28% reduction of BrdU-positive cells, respectively. The luzindole + melatonin + REMSD group had 426 ± 70 cells per SGZ, which corresponds to ~18% less BrdU-expressing cells with respect to the melatonin + REMSD group. However, the only significant difference was obtained with respect to the REMSD group (*P* < 0.043). In contrast, the melatonin + REMSD and the luzindole + melatonin + REMSD group showed an increase in the number of BrdU-positive cells as compared to the sleep deprivation group (Figure 3). Luzindole + REMSD was administered to observe whether melatonin concentrations may change in hippocampal tissue. The present findings of this group indicate that luzindole did not have a significant effect in melatonin levels. Thus, we decided to exclude group of the cell analysis to keep the use of animals as minimum as possible.

**Figure 4:**
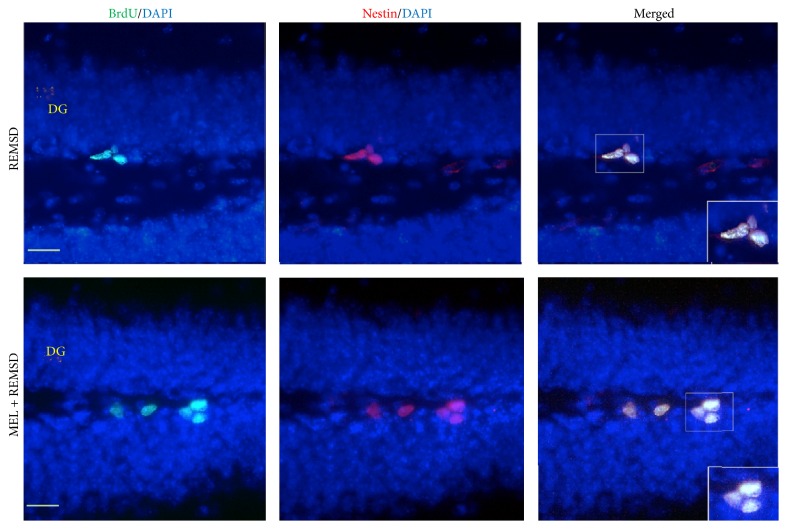
Representative immunofluorescence images of BrdU/nestin-expressing cells. BrdU and nestin expression in the subgranular zone of dentate gyrus (DG) of the sleep-deprived group (REMSD) and the MEL + REMSD group. Melatonin treatment increased the number of neural progenitors in DG as compared with controls. Bars = 10 *µ*m.

**Figure 5:**
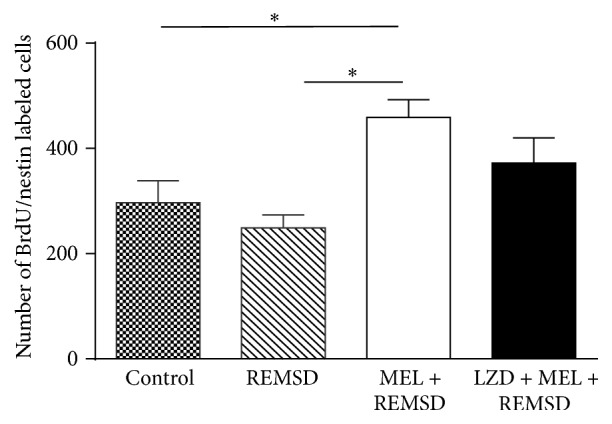
Number of BrdU/nestin label cells in dentate gyrus of hippocampi tissues. Bars express means ± SD. (*∗*) We found a statistical differences for control versus MEL + REMSD and REMSD versus MEL + REM sleep deprivation groups (^*∗*^ *P* < 0.021). Mann-Whitney *U* test.

To identify neuronal precursor cells in the SGZ, we colabeled our brain sections with anti-nestin antibody (Figure 4). The control group had 297 ± 4 cells per SGZ as compared to the melatonin + REMSD group (444 ± 3 cells per SGZ, *P* < 0.021). The melatonin + REMSD group also showed a statistical difference with the REMSD group (237 ± 24 cells per SGZ; *P* < 0.021). The luzindole + melatonin + REMSD group showed no significant differences as compared to the other groups (390 ± 48 cells) (Figure 5). In summary, the REMSD group had a decrease of 45.8% and the control group had a decrease of 33% in the number of progenitor cells when compared to the melatonin + REMSD group.

### 4.3. Western Blot of Antiapoptotic Proteins

**Figure 6:**
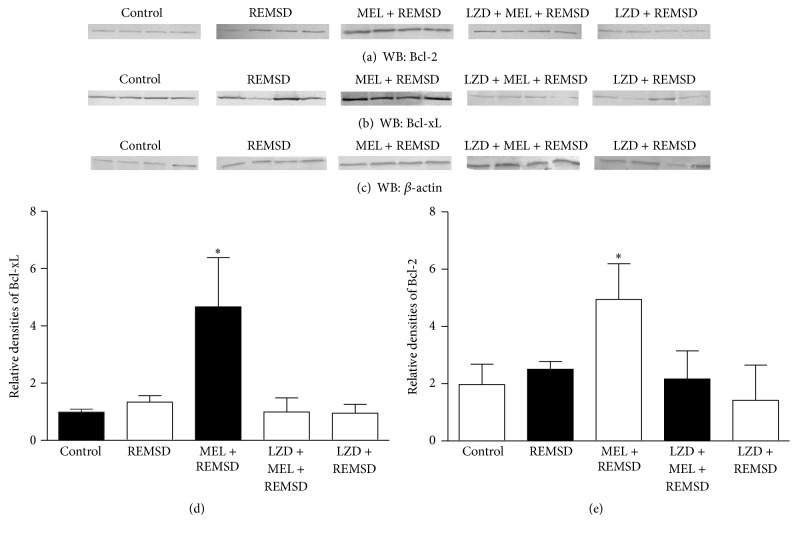
Western Blot analysis. (a) shows Bcl-2 qualitative protein expression. (b) shows Bcl-xL protein expression. (c) shows *β*-actin protein expression. Bars express mean ± SD. (*∗*) (d) Relative densities of Bcl-xL. (e) Relative densities of Bcl-2. Bcl-2 and Bcl-xL are overexpressed in the group with melatonin treatment with a significant difference versus the other groups (^*∗*^ *P* < 0.021, Mann-Whitney “*U*” test).

To analyze whether the changes in the number of SGZ progenitors were associated with antiapoptotic mechanisms, we analyzed the expression of Bcl2 and Bcl-xL (Figure 6). Our findings indicate that the expression of Bcl-2 protein in the melatonin group was significantly increased as compared to the other groups (*P* < 0.021) (Figure 6(a)). We also observed a significant increase in the expression of Bcl-xL in the group that received melatonin (*P* < 0.021; Figure 6(e)).

## 5. Discussion

Sleep deprivation is one of the most stressful agents with serious physical and psychological repercussions for sleep-deprived individuals. It has been postulated that one of the functions of REM sleep is to maintain longevity, survival, and integrity of neurons [14–16, 55–58]. Our hypothesis focuses on the possibility of generating a neuroprotective effect on neural precursors in the hippocampal SGZ by administrating melatonin [59]. This neuroindole is a pleiotropic molecule that is used to treat several pathologic conditions, which has well-known antioxidant properties and regulates the metabolism of neural cells via melatonin receptors, MAPK/ERK signaling, histone acetylation, neurotrophic factors, basic helix-loop-helix (bHLH) factors, and the nuclear factor erythroid 2-related factor 2 (Nrf2) [60–62].

Under conditions of sleep deprivation, 15 mg/kg melatonin reverses the levels of some oxidative stress markers, such as NO, MDA, and SOD activity [63]. Kumar and Singh demonstrated that melatonin treatment significantly restored the levels of glutathione, preserved catalase activity, and attenuated lipid peroxidation in 72 h SD mice [64].

To the best of our knowledge, this is the first study that explores the effects of the prophylactic administration of melatonin in REMSD conditions. Chronic administration of melatonin is able to disrupt circadian homeostasis without affecting animals' behavior [40, 41]. However, in our study, we cannot discard that REM sleep deprivation may contribute to developing anxiety-like behaviors by disrupting circadian rhythm but, even in that case, melatonin can ameliorate these stress levels as shown by previous reports [65–67]. Therefore, previous and current evidence help support the notion that melatonin may be considered as a possible therapeutic strategy when SD occurs [68].

Melatonin regulates circadian rhythms and promotes sleep via G protein-coupled receptors (MT1 and MT2) and nuclear receptors (RZR/RORa) in the suprachiasmatic nucleus [69, 70]. Besides, melatonin controls the flow of electrons by stopping the respiratory chain electron leakage and scavenging oxygen free radicals. These chemical properties appear to provide significant neuroprotection in neurodegenerative disease, neuroinflammation, and aging [71, 72]. We determined whether the prophylactic administration of melatonin by oral cannula modified the tissue levels of this hormone in the hippocampus of sleep-deprived animals. As expected the group that received oral melatonin had the highest concentration in the hippocampus as compared to the other groups. In contrast, the melatonin monotherapy did not modify the levels of this hormone in the brain tissue, nor the number of neuronal progenitors BrdU/nestin in the group of luzindole + melatonin + REMSD. The beneficial effects of melatonin might be mediated through MT1/MT2 receptors, because this neuroindole exerts its effects by modifying the mitochondrial homeostasis maintenance and preventing the expression of apoptotic genes [65, 73, 74].

Our findings indicate that REMSD decreases significantly the melatonin levels in the hippocampal tissue. A possible explanation for this event is that REMSD triggers the production of reactive oxygen or nitrogen species and lipid peroxidation, as well as decreases in the antioxidant system, such as glutathione and superoxide dismutase (SOD) [8, 48, 63, 66, 67]. REMSD may also impair the mitochondrial electron transport that can contribute to brain damage [75]. Consequently, melatonin can be rapidly consumed by interacting with free radicals generated by REMSD and if the intensity of the stressor is excessive, the amount of melatonin available in the brain tissue will be significantly reduced [29]. Hence, melatonin administration seems to be a feasible approach to restore endogenous tissue levels.

Recent findings showed that the sleep deprivation has a delayed effect on the cell differentiation in the SGZ and increases the apoptosis rate in the hippocampal CA1 and CA3 regions [76]. Our data indicate that exogenous administration of melatonin restores the tissue levels of this hormone in the hippocampus and increases the number of neural precursors and reveals a neuroprotective effect of melatonin against the deleterious consequences of REMSD. The beneficial effects of melatonin on SGZ precursors may rely on its capacity to increase the levels of phospho-c-Raf and phosphoextracellular signal-regulated kinase 1/2 (ERK1/2) through melatonin receptor [77]. Other studies have reported neuroprotective effect of melatonin in different animal models, including irradiation, aging, spinal cord injury, ischemia, hypoxia, and pinealectomy [34–37, 54, 77–80].

In addition, we explore the role of Bcl-2 and Bcl-xL that are critical regulators of programmed cell death [81]. After PSD, BAX is translocated to mitochondria that, in turn, decreases the membrane excitability of CA1 pyramidal neurons. Further evidence indicates that after 24 h of total SD the Bcl-2/Bax ratio decreases in the prefrontal cortex and pons [7, 82]. Melatonin can activate Bcl-2 by agonizing MT1 and MT2 receptors or by acting directly at the mitochondria level, where Bcl-2 coordinates the expression of pro- and antiapoptotic events [28]. Kuhn et al. using transgenic animals that expressed human Bcl-2 concluded that this protein promotes neuronal maturation and hippocampal neurogenesis in the adult brain [83].

In summary, our pharmacological approach showed that melatonin has neuroprotective effects against sleep deprivation. Nevertheless, future studies that analyze the antioxidant mechanism of melatonin on neural precursor cells and identify the cell type affected by sleep deprivation are required. Besides, the behavioral or cognitive consequences of melatonin treatment in REMSD conditions remain to be elucidated.

## 6. Conclusions

The prophylactic administration of melatonin increases the number of neural precursor cells in the adult SGZ. These effects are observed when melatonin is administered before and during REMSD. Interestingly, melatonin promotes an increase in the tissue levels of Bcl-2 and Bcl-xL of sleep-deprived animals. Taken together, our findings indicate that melatonin is an efficient neuroprotective agent against the noxious effects of REMSD.
